# Health care models guiding mental health policy in Kenya 1965 - 1997

**DOI:** 10.1186/1752-4458-4-9

**Published:** 2010-04-28

**Authors:** Florence A Muga, Rachel Jenkins

**Affiliations:** 1 Department of Psychiatry, University of Papua New Guinea, National Capital District, Papua New Guinea; 2 Department of Health Services and Population Research, Institute of Psychiatry, Kings College London, De Crespigny Park, London, UK

## Abstract

**Background:**

Mental health policy is needed to set the strategy and direction for the provision of mental health services in a country. Policy formulation does not occur in a vacuum, however, but is influenced by local and international factors in the health sector and other sectors.

**Methods:**

This study was carried out in 1997 to examine the evolution of mental health policy in Kenya between 1965 and 1997 in the context of changing international concepts of health and development. Qualitative content analysis of policy documents was combined with interviews of key policy makers.

**Results:**

The study showed that during the period 1965-1997 the generic health policy in Kenya changed from one based on the Medical Model in the 1960s and 1970s to one based on the Primary Health Care Model in the late 1970s and the 1980s and finally to one based on the Market Model of health care in the 1990s. The mental health policy, on the other hand, evolved from one based on the Medical Model in the 1960s to one based on the Primary Health Care Model in the 1990s, but did not embrace the Market Model of health care. This resulted in a situation in the 1990s where the mental health policy was rooted in a different conceptual model from that of the generic health policy under which it was supposed to be implemented. This "Model Muddlement" may have impeded the implementation of the mental health policy in Kenya.

**Conclusions:**

Integration of the national mental health policy with the general health policy and other sector policies would be appropriate and is now underway.

## Background

Mental health policy is needed to set the vision and overall strategy to address the mental health needs within a country, to set a framework for the provision of services and interventions and to give direction and benchmarks against which progress may be measured [1-3].

However, the context for mental health policy is complex and includes the general health policies, development polices, and perceived political demands and constraints. Studies in other developing countries such as India [4,5], Tanzania [6,7] and Nigeria [8,9] had demonstrated that the mental health policies in these countries had undergone changes over the decades, with the focus shifting from one policy to the next.

This paper reports a project carried out in 1997 to analyse the evolution of mental health policy in Kenya during the period 1965-1997, underlying health care models which informed both it and the generic national health policy, changing international concepts of development and health care; and the appropriateness of the policies in the context of Kenya as a low-income, developing country.

## Methods

Qualitative document analysis was combined with telephone interviews with key policy makers.

The key documents analysed included the Manifesto on African Socialism, which set the foundation for all Kenya's development policies; all the development plans between 1965 and 1997, which contained the country's development policies in all sectors including health; the Health Policy Framework of 1994, which defined the country's general health policy and the Mental Health Programme of Action of 1994 which defined the country's mental health policy. The policy makers selected for interview were the Director of Medical Services, as overall head of all health services in the country, the Director of Mental Health, as overall head of all mental health services in the country and a representative of the World Health Organization. These three policy makers were purposely selected because Kenya's mental health policy was set within the context of the country's general health policy but based on the principles of Primary Health Care as put forward by the World Health Organization.

The documents' context, content, themes and catchphrases were analysed in terms of their underlying conceptual health care models, namely the Medical Model, the Biopsychosocial Model and the Market Model.

The **Medical Model **[10-15] equates the improvement of health *"with the provision of medical care dispensed by growing numbers of specialists using narrow medical technologies for the benefit of the privileged few" *[15].

The Biopsychosocial Model [10,11,15,16]*"take [s] into account the patient, the social context in which he lives and the complementary system devised by society to deal with the disruptive effects of illness" *[11]. This Biopsychosocial Model is reflected in the concept of Primary Health Care which the World Health Organization defined as "*essential health care based on practical, scientifically sound and socially acceptable methods and technology made universally accessible to individuals and families in the community through their full participation and at a cost that the community and country can afford to maintain ..... bringing health care as close as possible to where people live and work, and constitutes the first element of a continuing health care process" *[15] Primary Health Care *"addresses the main health problems in the community, providing promotive, preventive, curative and rehabilitative services accordingly" *[15].

The **Market Model **[17,18], has been described as the anti-thesis of the principles of Primary Health Care [19], and "*assumes that health care is a product like any other. Where demand exists, then market forces will supply the goods required for a price.... in theory, the consumer is sovereign while the 'hidden hand' of market forces ensures the most efficient use of scarce resources by matching supply and demand" *[17]. It emphasizes individual, rather than community, responsibility for health care.

## Results

Three distinct historical periods were identified i.e. the first post-independence era (1965-1974), the second post-independence era (1975-1984) and the third post-independence era (1985-1997).

### The First Post-Independence Era (1965-1974)

#### The Context

The prevailing international concept of development was the modernization theory [20,21], which held that developing countries could catch up with the developed world through large-scale investments, with the benefits of an increased GNP 'trickling down' to the poor; while the international prevailing health model then was the Medical Model, introduced into developing countries from the industrialized countries, which emphasized specialized medical science and high technology as the means of eradicating disease. Kenya was newly independent and eager to catch up with the West, and had not only a relatively robust economy able to support the expansion of specialized health facilities but also doctors who, having been trained abroad, were very familiar and comfortable with the medical model.

#### Documentary analysis

The Manifesto on African Socialism [22], on which all development policies rested, had among its principles the need to draw on the best of African traditions and the country's willingness and desire to accept technical and financial assistance without strings The 1^st ^Development Plan [23] and the 2^nd ^Development Plan [24] focused on increasing national production and "accelerating the development of the economy even faster" [24] and on building specialized hospitals and training specialists. More emphasis was placed on curative services than on preventive services.

The government, considering health care provision to be essential and [23] committed itself to providing free health care for all Kenyans.

Kenya's generic health policy at this time was based on the Medical model as demonstrated by the policy emphasis on specialized, centralized, high technology facilities offering mostly curative care that was not universally accessible and did not involve the community in decision-making.

There was no explicit mental health policy during this era and mental health care was institution based, custodial and relied heavily on pharmacological treatment.

### The Second Post-Independence Era (1975-1984)

#### The Context

Following the failure of economic growth to reduce poverty and inequity in developing nations [25], a new approach to development was proposed, the satisfaction of an absolute level of basic needs [25].

Following the failure of the Medical Model to meet the health needs of the majority of the world's population, a new approach, the Primary Health Care strategy was proposed [15], which had as its main features equity, intersectoral collaboration, community participation and appropriate technology and which embraced a wider biopsychosocial approach to health as state of complete physical, mental and social well-being and not merely the absence of disease or infirmity [15].

Locally, Kenya's economy was deteriorating due to rising oil prices and falling market demands for Kenyan goods, the country had the world's highest population growth rate and the government, unable to expand specialized health services to meet the growing demand, was seeking alternative strategies for meeting the country's health care needs.

#### Documentary analysis

The policies of this era were stated in the 3^rd ^Development Plan [26], the 4^th ^Development Plan [27] and the 5^th ^Development Plan [28]. Despite the changed economic situation, the government still maintained it was responsible for providing health services in ample supply to all [27]. However, the government's emphasis shifted from a preoccupation with curative services to prevention; from training more doctors to training more nurses and medical assistants; and from building large centralized specialist hospitals to building smaller local district hospitals. Community participation was encouraged [26] and the importance of intersectoral collaboration was recognized [27] as was the role of traditional healers [27].

The health care model guiding Kenya's generic health policy during this period was the Primary Health Care model as shown by its explicit adoption of Primary Health Care and its emphasis on the specific Primary Health Care principles of community participation, intersectoral collaboration, promotive and preventive health care and the provision of health care by non-specialists.

Although Kenya had adopted the Primary Health Care strategy in 1979, recognized the importance of the mental dimension of health in the 4^th ^Development Plan [27] and added mental health as the ninth element of Primary Health Care in 1982, the country still had no explicit mental health policy during this era.

### The Third Post-Independence Era (1984-1997)

#### The Context

Internationally, following the global recession of the 1980s, there was a shift in developmental concepts away from the satisfaction of basic needs as a universal human right towards a market orientation in the provision of social services, including health. The World Health Organization continued to urge countries to formulate mental health policies and to provide mental health care along the principles of Primary Health Care [3,29], but the World Bank and the International Monetary Fund played an increasing role in determining the direction of the development policies of developing nations.

Critical of developing countries' attempts to treat health care as a right for their citizens and their attempts to provide free services to everyone [30], the World Bank advocated instead that these countries shift some of the health care costs from the state to the consumers. To this end, the World Bank and IMF exerted pressure on these governments, including Kenya, to introduce Structural Adjustment Programmes based on the market model. Locally, various factors such as the attempted coup d'etat of 1982, the drought of 1984, the suspension of donor aid in 1991 and the ever rising health demands of the population contributed to a decline in Kenya's economy.

#### Documentary analysis

The key policy documents of this period were the 6^th ^Development Plan [31], the 7^th ^Development Plan [32], the 8^th ^Development Plan [33], the Health Policy Framework of 1994 [34], the National Guidelines for the implementation of Primary Health Care [35], the Mental Health Act of 1989 [36] and the Mental Health Programme of Action of 1994 [37].

The key features of the development plans of this period were the increasing responsibility of the public and non-governmental agencies for health care, coupled with a decrease in the state's provision of health care. Market terms like "creating an enabling environment", "contracting", "budget rationalization", "cost-sharing" (ref 1989) crept into the policy documents. The 1989 development plan introduced Structural Adjustment [31]. Contrary to the government's previous commitment to bearing the cost of health care, cost sharing was introduced in public hospitals in 1989.

It was during this era that explicit mental health policies were formulated. The **National Guidelines for the implementation of Primary Health Care **of 1986 included a section detailing how mental health care could be incorporated into Primary Health Care. **The Mental Health Act **of 1989 legislated the decentralization of mental health services to the district level and their integration with general health services and also established a multi-sectoral Board of Mental Health to oversee the services, protect patient interests and advise the government on mental health matters. Following the passage of the Mental Health Act, work started in 1990 on formulating a **national mental health programme of action**. According to the national mental health programme of action, the objectives of the Mental Health Act of decentralization of mental health services and the demystification and de-stigmatization of mental illness were to be achieved through the integration of mental health with general health care, community participation in mental health care programmes and rehabilitation, and prevention of mental illness through early intervention by addressing precursors of mental illness as well as public mental health education. This policy was based on the principles of Primary Health Care.

Although work on the national programme of action had started in 1990, it was not published till 1994, by which time a new, overall health policy, the **Health Policy Framework **of 1994, [34] had also been published. This document Health Policy Framework was the over-riding health policy under which all health policies, including the mental health policy, had to be implemented.

The Health Policy Framework was a direct result of the Structural Adjustment process which had demanded Civil Service Reform, including Health Sector Reform. The main objectives of the Health Policy Framework were the reorganization of the Ministry of Health structure; the re-definition of the role of the Ministry of Health as creator of an enabling environment with a reduction in direct provision of health care, especially curative care' an increase in the role of other non-governmental health providers; an increased emphasis on alternative sources of health funding including insurance schemes and cost-sharing' the decentralization of health services and the re-orientation and deployment of personnel in line with the new policy.

Although the government's commitment to the Primary Health Care strategy was repeated, in reality the Health Policy Framework sounded like an echo of the 1987 World Bank Report, *Financing Health Services in Developing Countries: an Agenda for Reform *[30]. Table 1 compares some features of the two documents.

**Table 1:** Comparison between the World Bank policy document and the Kenyan policy document.

	WORLD BANK POLICY STUDY	KENYA HEALTH POLICY FRAMEWORK
Year Published	1987	1994
Problems identified in the health sector	inefficient spending on cost-effective health activities Internal inefficiency of public programmes *"lower-level facilities are underused, while. hospitals are overcrowded (p.3)"* Inequity in the distribution of benefits from health services	*"The Ministry of Health is seriously underfunded" (p.1)* *"unnecessary congestion of hospitals by patients who should be treated at lower cost in health centres and dispensaries"(p.1)* *"geographical disparities, which need to be addressed in order to achieve some equity" (p.4)*
4 Proposed policy reforms		
(Title/Catchphrase)	"Agenda for reform"	"Agenda for Reform"
Reform 1:	Charge user fees (p.3) *"The apparent willingness of households to pay at least some of the costs of health care" (p.3)*	*"raising of additional resources through widely accepted cost-sharing initiatives" (p.39)*
Reform 2:	Provide insurance or other risk coverage (p.4)	shift part of financial burden to insurance schemes (p.40)
Reform 3:	Use non-government resources effectively - government to focus on community/public health measures, rather than individuals (p.5) temporary government subsidies to NGO health providers (p.5)	Strengthening of NGOs, local authority, private and mission health service providers (p.37) enabling environment, which may include subsidies (p.37)
Reform 4:	Decentralize government health services - planning, budgeting and purchasing: allow revenue to be collected and retained close to the point of service delivery (p.6)	*"Further decentralization of planning, management and resource creation, control and use to the districts" *(p.36)

The model followed by the generic health policy during this period was the Market model espoused by the World Bank, as shown the similarities between the two documents above, and by the health policy document's emphasis on cost-effectiveness, budget rationalization and payment for health as a commodity.

The **telephone interviews **with the policy makers confirmed that the generic health policy was no longer based on the Primary Health Care model [38] that it had been influenced by the Structural Adjustment Programme and Health Sector Reform [38], that the mental health policy was still based on the Primary Health Care model but was facing pressure from donors and international agencies to conform to a market model [39] and that there was conflict between the generic health policy and the mental health policy [39].

## Discussion

The discussion will focus on the evolution of Kenya's health policy, the application of a market model to health and the appropriateness of Kenya's health policy in 1997. **Evolution of Kenya's health policy**. The international concept of development evolved from the Modernization Theory through the Basic Needs Theory to a Market based concept, while international health models likewise evolved from the Medical Model to the Biopsychosocial/Primary Health Care Model to a Market Model. In this context, and under international and domestic pressures, Kenya's generic national health policy evolved from the Medical Model through the Primary Health Care Model to the Market Model. However, the country's mental health policy evolved from the Medical Model to the Primary Health Care Model but did not embrace the Market Model despite pressure from the generic health policy to do so, leading to discordance between the two policies.

### The Market Model and Health

Contrary to the many assumptions which the Market model is based on [18,40-46], health is not a market commodity but a fundamental human right; consumers (patients) are not usually in a position to make rational choices about their needs or their provider; consumers do not necessarily use the commodity in the right quantities; the supply and demand will not necessarily balance out, since health needs are infinite and the supply in areas like mental health may never meet the ever-growing demand; the private sector may not necessarily cover what the government does not, especially in mental health care where insurance often excludes coverage of chronic 'pre-existing' mental illness thus making it an unprofitable area for the private sector; consumers, particularly in poorer nations, may not be able to pay for the services and in mental health the consumer may not even be the health seeker or the payer. How do you make someone pay to receive compulsory treatment by service providers he does not trust for a condition he does not believe he suffers from and which bothers other people rather than himself? Thus the market model is not a suitable one for health in general and mental health in particular.

### Appropriateness of Kenya's health policy

The overall health policy is inappropriate because it is based on the market model, which is inappropriate for health care in general. It is also inappropriate because it runs counter to Kenyan cultural values in that it promotes individualism or nuclear family-ism (as in insurance) while Kenyans rely on the extended family system as a key pillar of support. It also has an inappropriately narrow focus, in that while Kenyans practise a holistic biopsychosocial type of care that may also include pluralism, rebates are only given for allopathic biological care and not for alternative biological care e.g. by herbalists, not for alternative therapies by faith healers or traditional healers and not for social and psychological care provided by others. The policy is inappropriate in that it violates the principles of African Socialism the country's development is rooted in by overturning the state's stated obligation to provide medical care for all and by betraying its commitment only to accept financial assistance "without strings" [22]. Not only is the policy inappropriate in itself, but it is also inappropriate as an over-riding policy for the mental health policy to be implemented under, because the two policies are based on different models that are essentially anti-theses of each other, making it impossible for the two to be applied simultaneously. Not only were the two policies based on different models, but a parallel study carried out at the same time showed that Kenyan health workers who were supposed to be implementers of the policies still had as their frame of reference a Medical Model of health care [47].

Thus in 1997 a Primary Health Care based mental health policy was supposed to be implemented under the constraints of a Market based generic health policy by health workers who followed a Medical model of health care. These discrepancies resulted in a situation that has been described as "Model Muddlement" [48] and this may have hindered the implementation of Kenya's mental health policy.

## Conclusions

The findings showed that Kenya's health policy changed in response to influences from within and outside the country and these changes culminated in the adoption of a generic health policy that was inappropriate for the country.

Further review of the national mental health policy and its linkages with the general health policy and other sectors would be appropriate and is underway.

## Competing interests

The authors declare that they have no competing interests.

## Authors' contributions

FM carried out the research of the study and drafted the manuscript. RJ assisted in the writing of the paper. All authors read and approved the final manuscript.
